# Surface Covering of Downed Logs: Drivers of a Neglected Process in Dead Wood Ecology

**DOI:** 10.1371/journal.pone.0013237

**Published:** 2010-10-07

**Authors:** Mats Dynesius, Heloise Gibb, Joakim Hjältén

**Affiliations:** 1 Department of Ecology and Environmental Science, Umeå University, Umeå, Sweden; 2 Department of Zoology, La Trobe University, Melbourne, Australia; 3 Department of Wildlife, Fish, and Environmental Studies, Swedish University of Agricultural Sciences, Umeå, Sweden; Duke University, United States of America

## Abstract

Many species use coarse woody debris (CWD) and are disadvantaged by the forestry-induced loss of this resource. A neglected process affecting CWD is the covering of the surfaces of downed logs caused by sinking into the ground (increasing soil contact, mostly covering the underside of the log), and dense overgrowth by ground vegetation. Such cover is likely to profoundly influence the quality and accessibility of CWD for wood-inhabiting organisms, but the factors affecting covering are largely unknown. In a five-year experiment we determined predictors of covering rate of fresh logs in boreal forests and clear-cuts. Logs with branches were little covered because they had low longitudinal ground contact. For branchless logs, longitudinal ground contact was most strongly related to estimated peat depth (positive relation). The strongest predictor for total cover of branchless logs was longitudinal ground contact. To evaluate the effect on cover of factors other than longitudinal ground contact, we separately analyzed data from only those log sections that were in contact with the ground. Four factors were prominent predictors of percentage cover of such log sections: estimated peat depth, canopy shade (both increasing cover), potential solar radiation calculated from slope and slope aspect, and diameter of the log (both reducing cover). Peat increased cover directly through its low resistance, which allowed logs to sink and soil contact to increase. High moisture and low temperatures in pole-ward facing slopes and under a canopy favor peat formation through lowered decomposition and enhanced growth of peat-forming mosses, which also proved to rapidly overgrow logs. We found that in some boreal forests, peat and fast-growing mosses can rapidly cover logs lying on the ground. When actively introducing CWD for conservation purposes, we recommend that such rapid covering is avoided, thereby most likely improving the CWD's longevity as habitat for many species.

## Introduction

Decomposing wood, particularly tree trunks and other coarse woody debris, is important as habitat and resource for many forest organisms. CWD is in short supply in managed forests, compared with natural forests [1]–[5], resulting in a reduction in the abundance and diversity of wood-inhabiting organisms including arthropods, bryophytes, fungi, lichens, and vertebrates [6]–[8]. In response to this, managers conserve and create CWD during logging operations (e.g. [9]) and occasionally CWD is transported into forests to rehabilitate CWD-associated flora and fauna (for examples see [10]). Knowledge of the requirements of different wood-inhabiting organisms in terms of CWD qualities is accumulating, and the following factors have all proved important: position (lying vs. standing) [11], [12], site moisture [13], [14], stumps vs. slash [15], previous growth rate of the wood [16], superficial burning [17], [18], stand age and density [19], decay stage and log size [20], [21], sun-exposure [22], [23], and tree species [24]. The degree to which the surfaces of downed logs are covered, i.e. sink into the ground and/or are overgrown by ground vegetation, is also likely to affect wood-inhabiting organisms by changing the accessibility and characteristics of CWD. This covering process and its consequences are, however, almost totally unstudied. Here, we experimentally examine how properties of logs and the surrounding environment influence this covering process in boreal forests.

Several processes contribute to the covering of the surfaces of downed logs. Before the log has decomposed noticeably, it may sink into the soil due to gravity and ground vegetation may grow to densely cover log surfaces. Ground vegetation may overgrow a log either by diaspore establishment or by lateral overgrowing, i.e. when established vascular plants and bryophytes grow in from the sides [20]. Later on, decomposition successively reduces the structural integrity of the log, increasing soil contact. In addition, the increased soil contact along with the changed texture and height of the partially decomposed log facilitates overgrowing by ground-living bryophytes [20] and vascular plants.

Considering the extensive scientific literature on CWD, it is surprising that the variation in the rate of covering of log surfaces by sinking and overgrowing by ground vegetation and the factors controlling this rate have not been studied. Many studies have focused on the role of logs as substrate for bryophytes, lichens and vascular plants (e.g. [20], [25]–[31]), but these studies mostly focused on specialized CWD species and did not examine the sinking process. Harmon [26] and Kushnevskaya *et al*. [31] quantified cover by ground living species and/or litter on naturally occurring logs, but they considered only cover of the upper half of the log and did not include soil contact in their measure of cover. Although they did not test how cover related to environmental factors Kushnevskaya *et al.* [31] suggested that overgrowing may be affected by the type of ground vegetation present.

The aim of this study was, firstly, to experimentally quantify the variation in the rate of covering of the surfaces of introduced fresh logs by the combined action of sinking and laterally overgrowing ground vegetation after five growing seasons (six seasons for a small fraction of the logs) in boreal forests. Secondly, we assessed the degree to which rate of cover is slowed down by the presence of branches. Thirdly, we analyze how log characteristics and environmental factors affect the rate of covering of the surfaces of branchless logs. To further test the role of soil conditions, we identified understory vascular plant taxa (with known associations with site factors but not themselves participating in overgrowth) that grew disproportionally often around rapidly covered logs. To address the role of lateral overgrowing, we related the degree of cover of log surfaces to the presence of overgrowth and to the dominant overgrowth plant group.

## Materials and Methods

### Study area

We conducted the study in the Middle and North Boreal zone [32] in the provinces of Ångermanland and Åsele lappmark, northern Sweden (63°37′–64°17′ N). The mature forests in this region are dominated by Norway spruce (*Picea abies*) and Scots pine (*Pinus sylvestris*), with scattered broadleaved trees (mostly *Betula pubescens*, *Betula pendula*, and *Populus tremula*). Effective control of forest fires has been implemented for about 150 years.

### Study design

**Figure 1:**
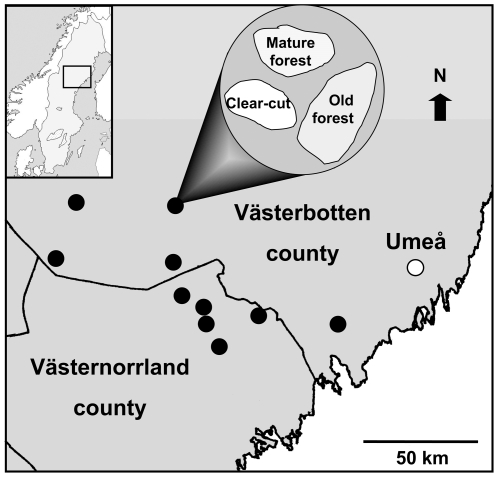
Location of the 10 study areas in Scandinavia. Each filled circle represents a study area that contains three sites (one old forest, one mature forest, and one clear-cut; right inset).

We used a large-scale experiment originally designed to assess the colonization of logs by wood-inhabiting organisms (see e.g. [12], [18], [33], [34]), explaining the somewhat complicated design described below. Almost half of the more than 2000 logs in the original experimental setup had not been disturbed by treatments (e.g. *in situ* superficial burning killing off ground vegetation) or by sampling (e.g. lifted to attach insect traps) and could therefore be used in the present study. The experiment setup included 10 localities containing three sites each; one clear-cut, one old forest in a reserve or national park (mean age c 160 years), and one unprotected mature forest (mean age c. 120 years) (Fig. 1). The forest stands, also those preceding the clear-cut, were all dominated by Norway spruce. The clear-cut sites were logged between 1999 and early 2001. Branchless logs of four meters in length were distributed in the 30 sites. Such logs are reasonable candidates for being transported into impoverished stands to rehabilitate CWD structures and wood-inhabiting communities in cases where felling of mature trees in the stand is not an option. The 4-m logs all originated from two logging operations, one performed early in 2001 (providing spruce logs distributed in one locality in early spring 2001, the “spruce locality” hereafter) and one in autumn 2001 (providing spruce and birch logs distributed in the other nine localities during the winter 2001/2002). Logs were distributed haphazardly and thus there should be no bias in the original quality of the logs between localities (except for the “spruce locality”) or among sites (clear-cut, mature forest, old forest) within localities. In each site, ten separate experimental blocks with seven logs each (five blocks with birch logs, *Betula* sp., and five blocks with Norway spruce) were established with the exception of the “spruce locality”, where only five blocks of spruce logs were distributed in each site (in total 1050 spruce logs in 150 blocks and 945 birch logs in 135 blocks). Six of the seven logs in a block were randomly distributed on the ground within the area (standardized to ∼20×20 m) assigned for the block, given the limitation that they had to be separated by 5 m. The seventh log was deliberately placed in the shade under a tree (shrubs on clear-cuts) within or in close proximity of the block. In addition to these four-meter logs taken from logging operations, 100 spruce trees were cut *in situ* at a height of three meters, thus creating a log with branches and top retained. One log of this kind was created in each of the spruce blocks in the 20 forested sites (not in clear-cuts).

The number of logs used in this study, i.e. logs not disturbed by treatments or sampling, varied from 68 to 111 among the nine localities with both spruce and birch (42 useful logs in the “spruce locality”), making up a total of 921 undisturbed logs. Out of these, 290 were in clear-cut sites, 310 in mature sites, 321 in old sites (Table 1 contains more details on local environments); 458 were spruce and 463 birch; and 37 spruce logs had branches and tops. The logs were initially selected to approximately produce a normal distribution with the mode at 20 cm. Out of the 921 logs used in this study, 77% had a diameter between 15 and 25 cm, 12% had diameters less than 15 cm (minimum 8.4 cm) and 11% had diameter of 25 cm or more (maximum 43.3 cm; Table 1).

**Table 1:** Diameter and local environments (predictors), and cover estimates (response variables) of the 921 logs used in the study.

	Mean	Median	Range	Explanations
**Predictors**				
Diameter of log (cm)	19.9	19.7	8.43–43.3	Mean of seven measurements per log
Altitude (m above sea level)	367	370	85–510	Taken from maps
PADIR (MJ cm^−2^ year^−1^)^a^	0.511	0.523	0.250–0.735	Potential Annual Direct Incident Radiation, calculated from ground slope, slope aspect and latitude
Estimated soil moisture	2.65	2.74	2.23–3.21	Highest indicator value among plant taxa recorded <0.5 m from the log
Estimated peat depth (cm)	23.6	13.7	4.6–70.7	Highest indicator value among plant taxa recorded <0.5 m from the log
Canopy shade	4.91	6	1–7	Seven classes representing increasing shade from open clear-cut far from forest edges (1) to shaded position under a spruce tree in closed forest (7).
**Response variables**				
Longitudinal ground contact (#)	4.24	4	0–7	Number out of 7 sampling points along the log that had direct ground contact. Estimate of the proportion of the length of the log that was in contact with the ground
Total cover (%)	23.1	19.3	0–94.3	Mean percentage of circumference covered (soil contact + ground vegetation cover), calculated from seven measurements per log, including those with zero cover. Estimate of the proportion of the entire log surface that was covered.
Cover of log sections in contact with the ground (%)^b^	36.1	34.0	2.2–95.9	Mean percentage of circumference covered at only those sampling points where the log had ground contact (1–7 measurements per log), thereby controlling for the effect of longitudinal ground contact on cover.

a Level ground has PADIR  = 0.522–0.529 MJ cm^−2^ year^−1^ within the latitude span of the sites.

b Only the 897 logs with ground contact at one or more sampling points of measure are included.

Because no long birch logs with tops and branches were present and because no such spruce logs lay on clear-cuts, we did not include the branched spruce logs in all analyses. Instead, we separately analyzed the difference in cover between these branched logs and the branchless spruce logs in their respective block. To attain a more balanced block design for some of the analyses, another 164 logs were excluded, resulting in a sample size of 720 logs (921 minus 37 branched logs minus 164 logs; labeled “reduced data set” in “Data analysis”). The excluded logs were single (in one case two) undisturbed logs in blocks where all the other logs had been disturbed. Hence, the reduced data set of 720 logs included only 122 blocks, whereas the full data set of 921 logs included 285 blocks.

### Data collection

Field data were collected from mid-September to mid-October 2006, i.e. five complete growing seasons after the logs were introduced (six in the “spruce locality”). The cover of log surfaces measured consisted of direct soil contact, mostly underneath, and cover by lateral overgrowing by dense mats of ground vegetation (i.e. bryophytes and vascular plants normally growing in undisturbed ground vegetation). Contribution to cover from overgrowth from colonizing seeds or spores or from log decomposition (as described in the introduction) were not addressed, because these processes operate slowly in the study area and do not add to cover of these fresh logs over these first few years.

Data on cover of log surfaces were obtained in the following manner. We located seven evenly distributed points on each of the branchless logs (0.5, 1.0, 1.5 m up to 3.5 m from one end of the 4-m log). For the logs with branches and tops, seven points were similarly distributed along their four basal meters. At each of the seven points, we measured stem diameter using calipers, and each measurement was used to calculate the circumference (assuming a circular form). At each point, we also recorded if the log had direct ground contact and, if so, measured the length of the circumference that was not covered by soil contact or ground vegetation using a measuring tape. The extent of cover was then calculated as the difference between the calculated total circumference and the measured portion of it that was not covered. Percentage cover was then calculated from covered circumference and total circumference for each point of measurement. At points without ground contact, percentage cover was set to 0. From these measurements we derived three response variables estimating different aspects of cover: *Longitudinal ground contact*, *Total cover*, and *Cover of sections in contact with the ground* (Table 1). Longitudinal ground contact was also used in predicting total cover.

Data on environmental conditions and log properties were also collected in the field, except for latitude and *altitude*, which were obtained from maps. For each log, we calculated mean *diameter* from the previously described seven measurements, and recorded *log species* (birch or spruce), presence/absence of branches and tops. We also recorded *canopy shade* (classes 1–7 where “1 and 2” represents logs placed in more or less exposed parts of clear-cuts, “3 and 4” in clear-cuts, but <20 m from forest edges, “5” in forests, but <10 m from a clear-cut edge, “6” in forests >10 m from clear-cut regardless of proximity of a shading tree, and “7” in a heavily shaded position under a spruce tree in forest). For each log, we also estimated local slope inclination (classes 0 (0–2.5), 5 (2.6–7.5), 10 (7.6–12.5) etc degrees, 90 degrees  = 100% slope) and slope aspect of the surrounding ground using a simple manual inclinometer and a compass. Potential annual direct incident radiation (*PADIR*) was then calculated for each log from slope inclination, slope aspect and latitude using equation 2 of McCune and Keon [35]. This is a globally applicable measure of the amount of solar radiation energy intercepted per year at ground level, had there been no clouds, trees or other shading structures. In this context, it reflects variation in temperature, moisture and light conditions among logs in each of the broad exposure types (forests and clear-cuts). PADIR attains high values on equator-facing slopes and low values on pole-ward facing slopes.

We estimated soil conditions under each log using indicator values of understory vascular plants growing within 0.5 m of the log as surrogates for soil moisture (*estimated soil moisture*) and peat depth (*estimated peat depth*). We chose this indirect method because it is simpler to perform compared to actual measurements, but also because it integrates temporally variable soil moisture conditions over time. Details on the procedures are found in Table S1. The collected presence data for understory vascular plants around each log was also used to identify taxa associated with rapid covering of log surfaces (see Data analysis).

Finally, we recorded presence/absence of lateral overgrowing of the logs by ground-living vegetation. This survey was conducted on a whole log basis and not by point sampling. If present, we identified the functional group(s) of plants that dominated the overgrowth, i.e. added most cover. The following three functional groups were used: (1) vascular plants, (2) *Sphagnum sp.* and *Polytrichum commune* (peat mosses and hair-cap moss), i.e. vertically growing, relatively large mosses typical of wet to moist ground, and (3) other mosses, i.e. horizontally growing mosses typical of mesic ground (mostly stair-step moss *Hylocomium splendens* and big redstem moss *Pleurozium schreberi*). In cases where no single group was clearly dominating the overgrowth, we recorded two or three groups as co-dominating.

### Data analysis

The differences in the response variables between spruce logs with tops and branches retained and 4-m spruce logs without tops and branches were assessed using all spruce logs from the 37 experimental blocks containing branched logs (37 branched and 215 branchless logs; only forested sites, not clear-cuts). Differences were tested using generalized linear models using a REML (residual maximum likelihood) approach in the statistical program JMP [36]. REML provides efficient estimates of treatment effects in unbalanced designs with more than one source of error. It differs from ordinary maximum likelihood estimation because it accounts for the degrees of freedom used in estimating treatment effects and is the most appropriate method for fitting mixed models [37], [38], [39]. The random factors “Locality”, “Site(Locality)” and “Block(Site(Locality))” were included in all models, but no *p*-values are provided for these factors using the REML method of analysis so they do not appear in the results.

We examined the effects of the other log properties and environmental factors on the three response variables for branchless logs using the reduced data set (720 logs, Table 2 and S2). Percentage data were arcsine-transformed before analysis. After transformation, data fulfilled the assumptions of the tests. We used three different approaches, assuring that interpretations were not biased by slight differences in the fit of different models. Firstly, we examined the fit of the full model (including all factors) using REML in JMP. Secondly, we tested all possible models (including from one to all factors) to find the model with the lowest Akaike Information Criterion [40] – the ‘best fit model’, using the function lme with the default REML method in the R computing environment [41]. To determine whether multicollinearity amongst predictor variables had influenced our results, we used a third statistical approach – hierarchical partitioning analysis [42], [43], on R. Hierarchical partitioning analysis was performed on means for each of the 30 sites because it cannot incorporate random factors. The analysis estimates the increase in model fit associated with each predictor variable by averaging its additional explanatory power in all models (i.e. all possible combinations of the predictor variables) in which that variable appears [44]. The explanatory power of each predictor variable is segregated into independent effects (I), i.e. those associated with that variable independently of other predictor variables and joint effects (J), attributable to the joint action of that variable with other predictors. Following the standard, we present only results for independent effects.

**Table 2:** Results from analyses of the effects of log properties and environmental conditions on longitudinal ground contact and our two measures of percentage cover by soil contact and ground vegetation, using 4-m branchless logs.

	Full GLM model		Best fit model^a^		Independent contribution	Correlation coefficient (r)^b^
	F	p	F	p	(%)	
**Longitudinal ground contact**	(*N = *720)		(*N = *720)		(*N = *30)	(*N = *720)
Estimated peat depth	28.44	<0.001	31.51	<0.001	28.5	0.25
Diameter	9.88	0.002	9.71	0.002	15	0.11
Log species	9.42	0.003	8.91	0.003	13.7	Birch > Spruce
Estimated soil moisture	4.30	0.039	3.94	0.048	8.4	0.12
PADIR	1.46	0.230	1.34	0.249	9.8	−0.11
Altitude	1.40	0.263			5.8	−0.02
Canopy shade	0.00	0.967			18.8	0.11
**Cover of log sections in contact with the ground (%)**	(*N* = 710^c^)		(*N* = 710^c^)		(*N* = 30)	(*N* = 710^c^)
Estimated peat depth	23.70	<0.001	23.70	<0.001	9.5	0.26
Diameter	22.19	<0.001	23.26	<0.001	7.3	−0.18
Log species	5.88	0.017	5.28	0.023	8.7	Birch>Spruce
PADIR	5.19	0.024	4.9	0.028	31.4	−0.26
Altitude	1.28	0.283			3.3	−0.09
Estimated soil moisture	0.29	0.593	0.22	0.636	9.1	0.22
Canopy shade	0.27	0.606	0.24	0.627	30.7	0.23
**Total cover (%)**	(*N* = 720)		(*N* = 720)		(*N* = 30)	(*N* = 720)
Longitudinal ground contact	1510.61	<0.001	1519.51	<0.001	71.2	0.83
Diameter	25.88	<0.001	27.15	<0.001	2.3	−0.03
Estimated peat depth	11.02	0.001	10.94	0.001	5	0.31
PADIR	6.61	0.011	6.65	0.011	6.3	−0.23
Log species	5.22	0.024	4.91	0.029	2.3	Birch > Spruce
Canopy shade	1.80	0.182	1,74	0.189	6.9	0.22
Altitude	0.30	0.597			1.9	−0.05
Estimated soil moisture	0.20	0.652	0.25	0.619	4.3	0.21

We used a set of 720 logs distributed over 122 experimental blocks in 30 sites. Results are from a full GLM model, a best fit model based on AICs, a hierarchical partitioning analysis (independent contribution), and a Pearson's correlation. The random factors “locality”, “site(locality)” and “block(site(locality))” were included in the full model and best model approaches, but do not show up in the table as a REML approach was used. Means from all logs within sites were used in the hierarchical partitioning analysis, because it cannot deal with random effects. Hence, this analysis only treats among-site (N = 30) variability in the response variables. The factors are sorted according to their F-value in the full model. Percentage data on cover were arcsine transformed before analysis. Longitudinal ground contact (0–7 points with contact) was not transformed. Degrees of freedom for predictors were 1 in all cases.

a Chosen using the Akaike Information Criterion [40].

b For tree species only qualitative relationships are presented, because this factor is binary and categorical.

c 10 logs did not have ground contact at any of the sampling points and could therefore not be used here.

To further analyze the role of soil conditions, we identified understory vascular plant taxa that grew mostly around logs with rapidly covered surfaces, but that did not themselves normally take part in any overgrowing. The habitat associations of the identified species were then used to infer site conditions conducive to covering by soil contact and overgrowing. The plant data used were the same as those used for estimated soil moisture and estimated peat depth (Table S1) and we used data from all 921 logs. Also, we included only the 10 most frequent of the 43 vascular plant taxa recorded to avoid accidental associations caused by low sample size. For each of these 10 taxa, we calculated mean longitudinal ground contact and mean percentage cover of sections in contact with the ground of all logs along which it appeared. In addition, we contrasted the percentage frequency of each taxon among all logs with its percentage frequency among (1) the 100 logs with the highest percentage cover of sections in contact with the ground (>54.89%) and (2) the logs with maximum longitudinal ground contact (all seven sampling points having ground contact, a set of 149 logs).

The role of lateral overgrowth was assessed by testing the hypotheses that the response variables *total cover* and *cover of sections in contact with the ground* score higher in the presence of overgrowing and that the effect was dependent on the identity of the dominant overgrowing functional group. To test these hypotheses we used REML models, with post-hoc Tukey's tests using data from the reduced data set on JMP.

## Results

**Figure 2:**
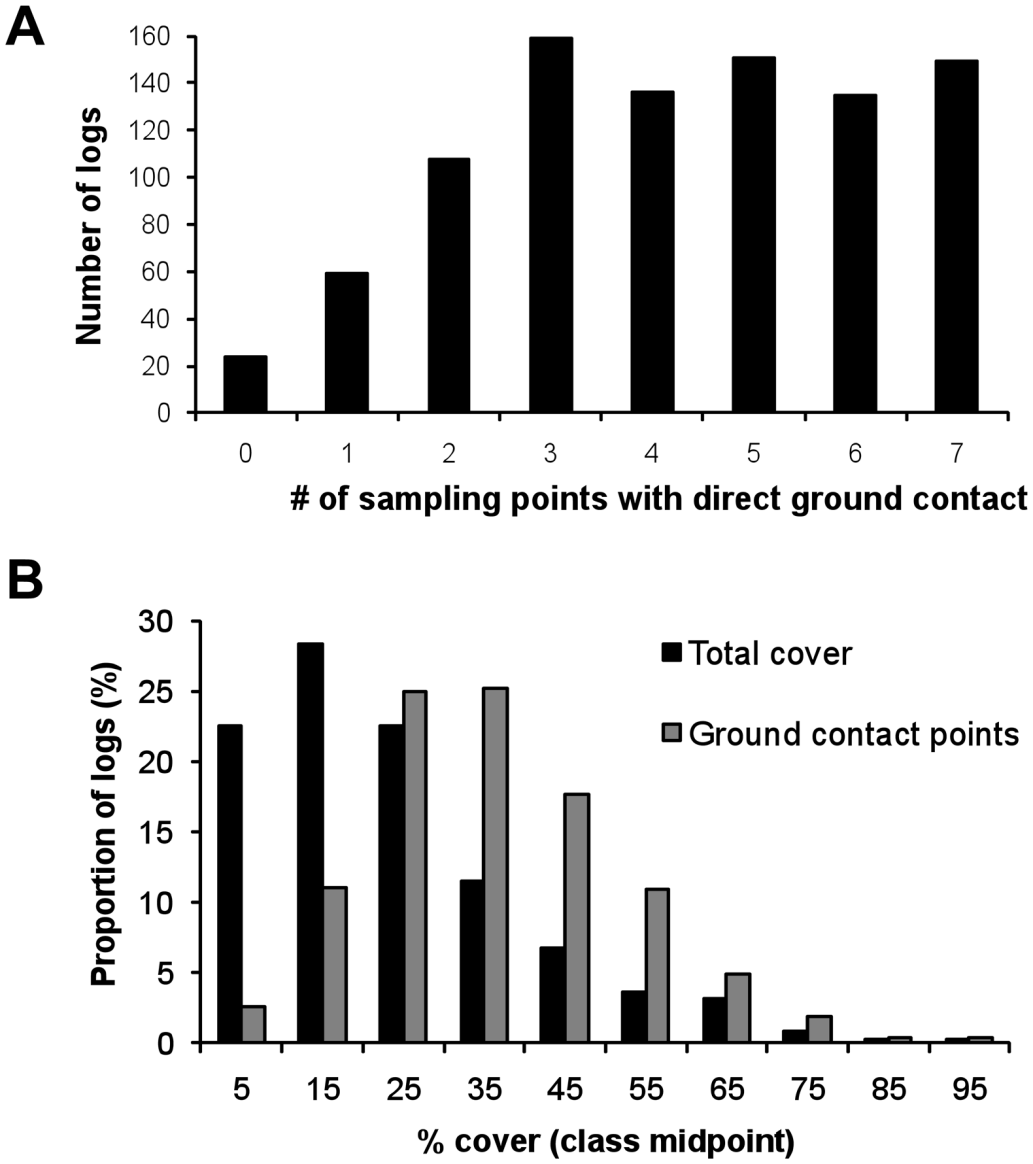
Frequency distribution of the experimental logs among classes of longitudinal ground contact and cover. (A) Longitudinal ground contact expressed as the number out of seven evenly spaced sampling points along each log that were in direct contact with the ground (921 logs). (B) Percentage of the circumference of logs that was covered by soil contact and ground vegetation after five years on the ground. Black bars represent the frequency of logs based on the mean cover of all seven sampling points for a log (total cover, 921 logs). Grey bars are the frequency of logs based on the mean cover at only those sampling points along a log that had ground contact (897 logs because 24 logs were excluded since they did not have ground contact at any of the seven sampling points).

The mean longitudinal ground contact of logs was 60% (Table 1). Few logs had less than a quarter of their length in contact with the ground, whereas higher scores were relatively evenly distributed (Fig. 2A). Mean total cover was close to one quarter, but a few logs were almost entirely covered. The frequency distribution was skewed with a median total cover of less than a fifth (Table 1, Fig. 2B). Mean cover of log sections in contact with the ground was more than one third.

The continuous predictors covered a wide range of values (Table 1), which was beneficial for the evaluation of their influence on the covering process. The relationships among predictors were generally weak (Table S2), but “estimated soil moisture” and “estimated peat depth” showed a strong positive correlation.

### Factors influencing longitudinal ground contact

**Figure 3:**
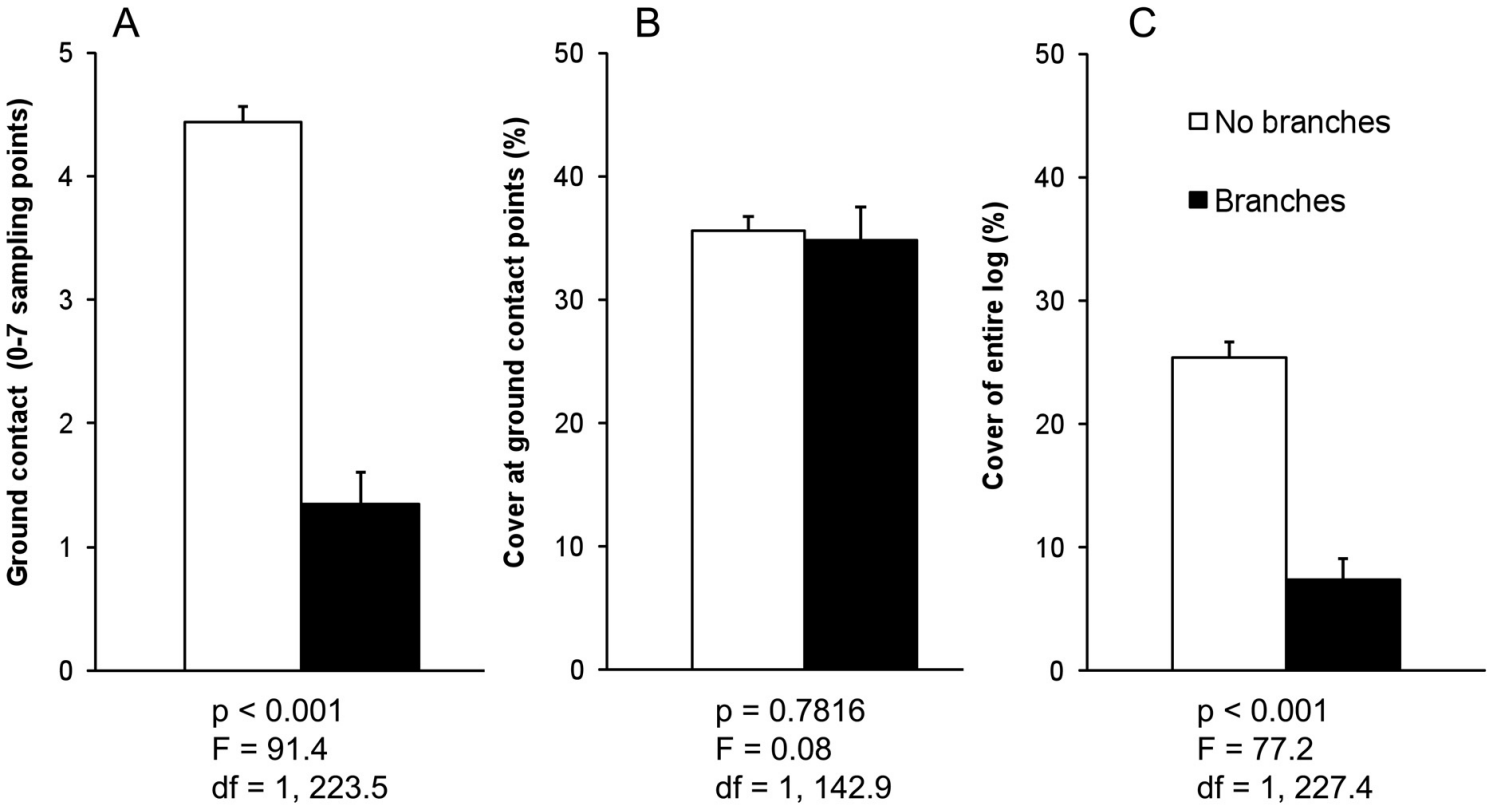
Longitudinal ground contact and cover of logs with and without branches and tops. (A) Longitudinal ground contact expressed as the number out of seven evenly spaced sampling points along each log, that were in direct contact with the ground. (B) Percentage cover by soil contact and ground vegetation for log sections in contact with the ground (mean of 1–7 measurements per log). (C) Percentage total cover of logs (mean of all 7 measurements per log). Results of the REML models are presented under the graphs. Black bars describe means and SE for long spruce logs with branches and tops (*N* = 37 in A and C, but only 24 in B because 13 logs did not have ground contact at any of the 7 sampling points) and open bars depict means and SE for all 4-m spruce logs without branches and tops from the same experimental blocks as the branched logs (*N* = 215 in A and C and 139 in B).

Longitudinal ground contact averaged 4.4 of the seven measurement points for the 4-m, branchless spruce logs, but only 1.6 for the long spruce logs with their branches and tops retained (Fig. 3A).

For the 4-m logs without branches, the strongest predictor of longitudinal ground contact was estimated peat depth, which increased ground contact (Table 2). Diameter of log (increasing contact) and tree species (birch having more contact than spruce) both contributed considerably to the full model and the best model. All these three factors also had relatively high independent contributions in the Hierarchical Partitioning Analysis summarizing models based on site means (Table 2), but shade from canopy appeared as an additional important factor (increasing cover). The importance of this relationship was probably accentuated by taking site means for both predictor and response variables because sites were characterized by being either high shade (forests) or low shade (clear-cuts). Taking means at the site level meant that the within-site variation was lost, but this was unavoidable because Hierarchical Partitioning Analysis is unable to deal with random factors.

### Factors influencing cover

Cover of log sections in contact with the ground was approximately the same for the branchless spruce logs and the spruce logs with their branches and tops retained (Fig. 3B). Thus, the considerably higher total cover found for branchless logs (Fig. 3C) was caused by their higher longitudinal ground contact.

For the branchless logs the most important predictor of total cover was clearly longitudinal ground contact (increasing cover; Table 2). When longitudinal ground contact was factored out by assessing only cover of log sections in contact with the ground, four factors were prominent predictors. Estimated peat depth (increasing cover) and diameter of log (reducing cover, unlike the increase in longitudinal ground contact with diameter) contributed most in the full and the best fit models. The Hierarchical Partitioning Analysis suggested that potential input of solar radiation (PADIR, higher cover on pole-ward facing slopes), and canopy shading (increasing cover) clearly contributed most to all possible models. Again, it is important to note that this result was based on site means. In the full and the best fit models, which incorporated the full variation in the data set, PADIR was only moderately important and canopy shading was unimportant (Table 2). For logs deliberately placed under spruce trees in forests (the most shaded position), cover of log sections in contact with the ground was lower than in randomly selected forest settings (34.5% vs. 39.2%).

### Vascular plants associated with high longitudinal ground contact and cover

Cloudberry (*Rubus chamaemorus*), wood horsetail (*Equisetum sylvaticum*), and the sedge *Carex globularis* were the three species with the strongest association with both high longitudinal ground contact and high cover of log sections in contact with the ground (Table S3). These three species are all typical of moist to wet, peat-forming sites (Table S1).

### Lateral overgrowth

**Figure 4:**
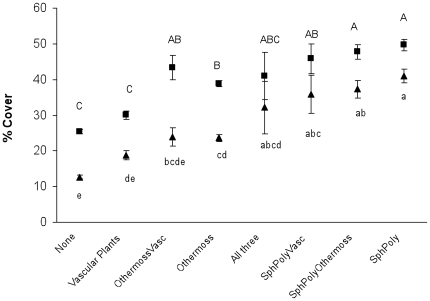
Percentage cover of logs having no vegetation overgrowth or having different kinds of overgrowth. “None” denotes the logs with no overgrowth. The logs having overgrowth are grouped according to the plant group(s) that dominated their overgrowth: vascular plants (mostly grasses and dwarf shrubs), *Sphagnum* and/or *Polytrichum* mosses (“SphPoly”; peat-forming mosses growing on moist ground), other mosses (species of less moist ground) and four groups with co-dominance by different combinations of these three types. Triangles denote mean percentage total cover (720 logs; summarizing all seven sampling points per log) and squares indicate mean percentage cover of log sections in contact with the ground (summarizing 1–7 sampling points per log depending on the degree of longitudinal ground contact; 710 logs since 10 logs did not have ground contact at any sampling points). Bars depict standard error. Results from REML model: *p*<0.001, *F* = 35.31, df = 7, 670.6 (total cover) and *p*<0.001, *F* = 29.30, df = 7, 666.7 (log sections in contact with the ground). Different letters indicate significant differences between groups (Tukey's test, α = 0.05).

Ground vegetation had grown to cover some portion of the sides (or even upper parts) on 615 of the 921 logs examined (67%), constituting a similar proportion of the logs in forests and clear-cuts. Overgrowing was predominantly by vascular plants for 116 logs, by *Sphagnum* and/or *Polytrichum* for 153 logs, and by other mosses for 237 logs. Different combinations of these three functional groups co-dominated on 109 logs. Logs mostly overgrown by peat mosses *Sphagnum* sp. and/or hair-cap moss *Polytrichum commune* were significantly more covered than both those not overgrown at all and those overgrown predominately by other mosses or by vascular plants (Fig. 4). On clear-cuts, dense mats of the basal parts of wavy hairgrass (*Deschampsia flexuosa*), bilberry (*Vaccinium myrtillus*), lingonberry (*Vaccinium vitis-idaea*), and also sometimes the sedge *Carex globularis*, covered the sides of some logs. In forests, vascular plants rarely dominated overgrowth.

## Discussion

The main aim of the study was to identify factors controlling the rate of covering of downed logs by soil contact and ground vegetation. Here, we discuss the mechanisms by which the identified factors may affect cover, but we also consider the potential effects on wood-inhabiting organisms of such cover and the implications of the results for conservation management of forests. The mechanisms and effects discussed may also be applicable to other forest ecosystems where peat and/or thick moss carpets are present, for example in tropical peat swamp forests [45]. In regions where peat and fast-growing ground mosses are scarce, sinking and overgrowth are, however, likely to be unimportant factors in dead-wood ecology.

### Factors influencing cover

Cover was influenced by the presence of branches, log diameter, peat depth, tree species and factors related to sun exposure. The strong influence of branches on longitudinal ground contact and thus cover (Fig. 3) is easily interpreted: branches support logs in a position above the ground [46], thereby effectively delaying both sinking and lateral overgrowing. The increase in longitudinal ground contact associated with increased log diameter is likely caused by the higher load per unit area of thicker logs, making them sink more readily. However, a log with a larger diameter also has a larger surface area to be covered, explaining our finding that percent cover of log sections in contact with the ground decreased with diameter (Table 2). The higher longitudinal ground contact found for birch logs may also be a result of the higher load per unit area. Birch probably sunk into the ground faster because birch wood is denser than spruce wood (e.g. [47]).

Sinking into low-resistance peat soil is likely the most important explanation for the increase with estimated peat depth in both longitudinal ground contact and percentage cover of log sections in contact with the ground. Peat also reduces micro-topography by filling depressions in the mineral soil, further promoting ground contact. Furthermore, Bisbee *et al.* [48] found that peat depth is positively related to bryophyte production in boreal forests. Common fast-growing and peat-forming boreal forest bryophytes include *Sphagnum* mosses [48] and large species of *Polytrichum* mosses (mostly *P. commune*). In addition to the peat formed by these mosses, the thick and loose carpets of living mosses facilitate sinking. Their high growth rate also explains their important role in overgrowing that we demonstrate. Thus, deep peat is associated with both sinking and overgrowing by ground-living mosses. Peat depth was, however, estimated from the presence of plant species and not measured directly, likely leaving some variation in peat depth unaccounted for. Therefore, the predictive power of some other factors in the models may be partly due to their effect on peat formation. This is probably the case for canopy shade (increasing longitudinal ground contact and percentage cover of log sections in contact with the ground) and PADIR (higher percentage cover of log sections in contact with the ground in pole-ward facing slopes), which both show up as important factors in the models. Peat is more rapidly formed under cold and wet conditions and both temperature and moisture are undoubtedly affected by input of solar energy.

Because laterally overgrowing plants need light, lateral overgrowth should decrease with canopy shade and increase with PADIR. Our results show, however, that vascular plants were the main beneficiaries of the abundant light on clear-cuts. For morphological reasons, boreal field layer vascular plants should be slower over-growers than *Sphagnum* and *Polytrichum*. These mosses grow in a cushion-like fashion, with most of each year's growth increment adding to overgrowth. Most boreal vascular plants do not form such cushions, and much of the aboveground biomass dies off each year and decomposes. Often, only the growth of the basal parts remains to effectively cover the log the following year. In addition, average temperature is higher and surface and air moisture lower on clear-cuts and in equator-facing slopes, potentially reducing moss growth. Also, night temperatures are lower in clear-cuts, a factor known to retard *Sphagnum* growth [49]. However, at very low light levels, such as those found under dense spruce trees in forests, we found cover to be lower, probably as a result of low light levels causing low growth rates of ground-living mosses. This interpretation is consistent with the finding that *Sphagnum* cover on the floor of mature boreal forests increases with canopy openness [48], [50]. The coarse tree roots close to spruce trees may also be involved in the low cover under trees, by stabilizing the soil and thereby preventing sinking.

Boulders and rocks cause small-scale topographic heterogeneity in forests. Such heterogeneity reduces sinking and ground contact and should thus reduce cover rate. This factor was not evaluated in our study, but its importance has previously been shown. Ódor and van Hees [51] found that in Hungarian beech forests, thin logs could function as substrate for bryophytes specialized on CWD only when boulder cover was high. In such forests, thin logs often lies in elevated positions and are therefore covered less rapidly by dead leaves.

### Implications for wood-inhabiting organisms

The effects on wood-inhabiting organisms of surface covering of logs by soil contact and ground vegetation remain almost completely unknown, but habitat properties such as moisture, temperature, CO_2_, oxygen, and light are undoubtedly affected. Obvious examples of effects include small specialized wood-inhabiting photosynthesizing organisms, e.g. liverworts and lichens, succumbing to light deficit under thick cover, and fungi that are unable to form fruiting bodies on substrates other than uncovered CWD. In addition, the assemblages of insects and fungi differ between logs with branches and tops and logs without [18], [52], and at least part of this difference may be attributed to differences in cover including soil contact. For thermophilous species, the cool environment of a covered log should be less suitable, whereas moderately raised moisture levels stimulate fungal growth and thus also the abundance of fungivores [34], [53], [54]. Wet conditions, on the other hand, may cause anoxia with severe effects for aerobic wood-inhabiting species. Stabilized moisture and temperature conditions would, of course, promote species with narrow tolerances.

Conditions for colonization must also change. Organisms present in or on the ground should more readily colonize a log with much ground contact, e.g. mosses and vascular plants. Examples of this facilitation also include certain soil-living mycorrhizal fungi, which preferentially or exclusively form fruiting bodies on CWD. However, species more specialized on CWD are not usually present in or on the ground, but need to colonize from other CWD that is in direct contact or from more distant CWD through passive wind dispersal (e.g. fungi, liverworts, mosses) or active movements, mostly flight (e.g. insects). For these organisms, ground contact will be of little or no help in colonization. Instead, soil contact and dense ground vegetation cover may function as a barrier to establishment when spores of wood-inhabiting fungi settle, and wood-inhabiting insects may potentially be hindered in their oviposition. Further studies on the effects of cover on the accessibility and quality of CWD would significantly enhance our ability to target CWD restoration to effectively conserve CWD-associated biodiversity.

### Management implications

The practice of creating dead wood to satisfy biodiversity targets is becoming increasingly common, worldwide [9], [10]. Given the obvious negative effects of rapid covering on some wood-inhabiting organisms (e.g. liverworts), managers actively introducing CWD on the ground of boreal forests should take special considerations in north-facing slopes and where peat and/or large and fast-growing mosses are present. We recommend that under these circumstances ground contact is reduced by retaining branches and tops, a practice that could also be advocated because it is more similar to naturally falling trees or snags. However, if for some reason trees could not be felled *in situ*, it is much more difficult to transport logs with branches and tops than without. Placing logs on projecting features such as stumps, logs, hummocks, tree bases or small boulders would provide a similar effect to leaving branches. Placement on existing logs and stumps may also facilitate colonization by species specialized on CWD, although care should be taken to avoid destruction of these substrates.

Avoiding moist places altogether would, however, be severely counterproductive; many wood-inhabiting species thrive and/or are restricted to moist habitats, because of the stable microclimate, high humidity and/or low temperature. For example, the abundance and species richness of wood-inhabiting bryophytes are higher in relatively moist and sheltered settings such as forests compared with clear-cuts [55], stream-side compared with upland forests [13] and pole-ward facing compared with equator-facing slopes [14].

## Supporting Information

Table S1 List of the vascular plant taxa used as indicators of the peat depth and soil moisture under each log and their respective indicator values.
(0.05 MB DOC)

Table S2 Relationships among the seven predictor variables.
(0.04 MB DOC)

Table S3 Vascular plant taxa recorded within 0.5 m from greater than 5% of the 921 logs and their association with cover rate of logs.
(0.05 MB DOC)
