# Supplementary material for: Surface Covering of Downed Logs: Drivers of a Neglected Process in Dead Wood Ecology
Source: PLoS One. 2010 Oct 7;5(10):e13237. doi: 10.1371/journal.pone.0013237 (PMC2951364; doi:10.1371/journal.pone.0013237)
Supplement: Table S1 — List of the vascular plant taxa used as indicators of the peat depth and soil moisture under each log and their respective indicator values. (0.05 MB DOC) [file pone.0013237.s001.doc]

**Table S1. List of the vascular plant taxa used as indicators of the peat depth and soil moisture under each log and their respective indicator values.**

| Taxa* | Mean peat (cm) | Mean moisture |
| --- | --- | --- |
| *Eriophorum vaginatum* | 70.7 | 3.05 |
| *Molinia caerulea* | 66.7 | 2.92 |
| *Carex echinata* | 61.8 | 3.21 |
| *Rubus chamaemorus* | 54.9 | 3.00 |
| *Potentilla erecta* | 45.7 | 2.75 |
| *Viola epipsila* | 45.7 | 2.97 |
| *Filipendula ulmaria* | 37.6 | 3.00 |
| *Ledum palustre* | 29.6 | 2.58 |
| *Calamagrostis* sp. | 29.2 | 2.87 |
| *Moneses uniflora* | 28.5 | 2.75 |
| *Carex globularis* | 26.6 | 2.86 |
| *Equisetum pratense* | 23.1 | 2.92 |
| *Equisteum sylvaticum* | 19.2 | 2.88 |
| *Deschampsia caespitosa* | 17.0 | 2.74 |
| *Cornus suecica* | 15.2 | 2.58 |
| *Orthilia secunda* | 12.9 | 2.71 |
| *Geranium sylvaticum* | 12.2 | 2.59 |
| *Rubus saxatilis* | 12.0 | 2.57 |
| *Dryopteris carthusiana/expansa* | 11.5 | 2.62 |
| *Thelypteris phaegopteris* | 11.2 | 2.94 |
| *Agrostis sp.* | 11.1 | 2.49 |
| *Lactuca alpina* | 11.0 | 2.80 |
| *Viola riviniana* | 10.8 | 2.36 |
| *Melica nutans* | 10.7 | 2.52 |
| *Carex digitata* | 10.0 | 2.39 |
| *Athyrium filix-femina* | 9.6 | 2.77 |
| *Fragaria vesca* | 8.0 | 2.42 |
| *Oxalis acetocella* | 7.9 | 2.54 |
| *Gymnocarpium dryopteris* | 7.8 | 2.51 |
| *Maianthemum bifolium* | 6.9 | 2.39 |
| *Anemone hepatica* | 5.2 | 2.39 |
| *Goodyera repens* | 4.6 | 2.23 |
| *Veronica officinalis* | 3.6 | 2.28 |
| *Milium effusum* | 3.2 | 2.63 |

No published list of indicator values of plant taxa for northern Sweden was available, but indicator values for soil moisture were provided by an ongoing project (Ursula Zinko pers. comm.) We used the same data source and method to calculate indicator values for peat depth. The data source was an extensive North Swedish data set from the Swedish NFI (National Forest Inventory), including both soil and plant data from the same plots. The indicator values for each taxon were calculated as the mean value of the measured peat depth/estimated soil moisture class from all the NFI plots where the taxon was present. Soil moisture in the Swedish NFI is a coarse visual estimate in five classes related to the average depth of the ground water table during the vegetation period: Class 1 denotes dry soils (> 2m to average ground water table), 2 mesic (> 1 m), 3 mesic to moist, 4 moist and class 5 denotes wet soils. Peat depth is measured by NFI down to 99 cm. Plots with >99 cm peat were given the value 99 cm in our analysis.

We did not record all understory taxa growing close to the logs in our study. For example, the most frequent taxa in the region were excluded, because we assumed that their high frequency means that they occur under a wide range of soil conditions. Excluded taxa are listed in the footnote below the table. We recorded a total of 43 taxa, but nine of these were not present in the NFI data and could thus not be assigned an indicator value. The highest indicator value among the taxa recorded close to a log was used as surrogate for soil moisture/peat depth for that log. When none of the 34 taxa were present (35% of the logs), the log was assigned a “null” value: the indicator values of the most common species in the region, lingonberry (*Vaccinium vitis-idaea*, peat: 13.7, moisture 2.32), which appeared in 86% of the NFI plots.

* Although present around logs, the following understorey species were not recorded and thus not used as indicators: *Calluna vulgaris*, *Carex vaginata,* *Dactylorhiza* sp*., Deschampsia flexuosa*, *Empetrum* spp., *Epilobium angustifolium*, *Linnaea borealis*, *Listera cordata, Luzula pilosa, Lycopodium annotinum,* *Melampyrum pratense, M. sylvaticum, Rubus idaea, Solidago virgaurea, Trientalis europaea*, *Vaccinium myrtillus*, *V. oxycoccus, V. uliginosum*, and *V. vitis-idaea*.
