# Supplementary material for: Surface Covering of Downed Logs: Drivers of a Neglected Process in Dead Wood Ecology
Source: PLoS One. 2010 Oct 7;5(10):e13237. doi: 10.1371/journal.pone.0013237 (PMC2951364; doi:10.1371/journal.pone.0013237)
Supplement: Table S2 — Relationships among the seven predictor variables. (0.04 MB DOC) [file pone.0013237.s002.doc]

**Table S2.** Relationships among the seven predictor variables.

|  | Diameter | Altitude | PADIR | Moisture | Peat | Tree species | |
| --- | --- | --- | --- | --- | --- | --- | --- |
|  |  |  |  |  |  | Birch | Spruce |
|  | r | r | r | r | r | Mean | Mean |
| Diameter | 1 |  |  |  |  | 19.0 | 21.3 |
| Altitude | 0.184 | 1 |  |  |  | 384 | 352 |
| PADIR | -0.040 | -0.034 | 1 |  |  | 0.514 | 0.508 |
| Indicated soil moisture | -0.006 | -0.165 | -0.059 | 1 |  | 2.64 | 2.67 |
| Indicated peat depth | 0.085 | -0.079 | -0.089 | **0.775** | 1 | 22.6 | 24.2 |
| Canopy shade | -0.027 | 0.099 | -0.174 | 0.097 | 0.124 | 4.92 | 4.87 |

Correlations among six of the predictors used in the analyses presented in Table 2 (Pearson correlation, N=720, correlations with r > 0.75 are marked with bold correlation coefficients). For the binary categorical variable “Tree species” means for these six variables are presented instead of correlation coefficients.
