# Supplementary material for: Surface Covering of Downed Logs: Drivers of a Neglected Process in Dead Wood Ecology
Source: PLoS One. 2010 Oct 7;5(10):e13237. doi: 10.1371/journal.pone.0013237 (PMC2951364; doi:10.1371/journal.pone.0013237)
Supplement: Table S3 — Vascular plant taxa recorded within 0.5 m from greater than 5% of the 921 logs and their association with cover rate of logs. (0.05 MB DOC) [file pone.0013237.s003.doc]

**Table S3.** Vascular plant taxa recorded within 0.5 m from greater than 5 % of the 921 logs and their association with cover rate of logs. The mean cover and ground contact of all 921 logs are given for comparison (row five). Vascular plant species with higher values than for all logs are associated with faster covering of log surfaces than average and *vice versa*.

| Taxon | Cover of log sections in contact with the ground (%)* | | Longitudinal ground contact (%) | | Proportion of all logs | Proportion of logs with maximum ground contact | Proportion of logs with highest cover of log sections in contact with the ground |
| --- | --- | --- | --- | --- | --- | --- | --- |
|  | Mean | SE | Mean | SE | % | % | % |
| *Rubus chamaemorus* | 45 | 1.57 | 74 | 2.58 | 13 | 27 | 32 |
| *Carex globularis* | 41 | 0.83 | 64 | 1.45 | 45 | 76 | 66 |
| *Equisteum sylvaticum* | 41 | 1.19 | 64 | 2.02 | 23 | 40 | 34 |
| *Orthilia secunda* | 39 | 1.49 | 62 | 2.55 | 12 | 20 | 11 |
| All logs | 36 | 0.52 | 61 | 0.92 | - | - | - |
| *Calamagrostis* sp. | 35 | 2.22 | 60 | 3.87 | 6 | 6 | 6 |
| *Dryopteris carthusiana/expansa* | 34 | 1.44 | 55 | 3.16 | 9 | 5 | 5 |
| *Gymnocarpium dryopteris* | 33 | 0.97 | 55 | 1.77 | 26 | 17 | 20 |
| *Maianthemum bifolium* | 32 | 1.83 | 55 | 3.59 | 6 | 4 | 2 |
| *Geranium sylvaticum* | 30 | 1.53 | 56 | 3.55 | 6 | 0 | 4 |
| *Oxalis acetocella* | 29 | 1.27 | 51 | 2.82 | 9 | 2 | 4 |

For each taxon, mean and standard error on cover and ground contact is presented for the logs with which they co-occurred (mean and standard error, first two columns). In the remaining three columns, the number of logs with which the taxon co-occurred is presented as proportions (%) of three groups of logs: (i) all 921 logs, (ii) the 149 logs with maximum longitudinal ground contact, i.e. those with all seven sampling points in contact with the ground, and (iii) the 100 logs with > 54.89% cover of log sections in contact with the ground. The taxa are sorted with those associated with logs having high cover first. The following understorey taxa were (although sometimes present) not recorded: *Calluna vulgaris*, *Carex vaginata,* *Dactylorhiza* sp*., Deschampsia flexuosa*, *Empetrum* spp., *Epilobium angustifolium*, *Linnaea borealis*, *Listera cordata, Luzula pilosa, Lycopodium annotinum,* *Melampyrum pratense, M. sylvaticum, Rubus idaea, Solidago virgaurea, Trientalis europaea*, *Vaccinium myrtillus*, *V. oxycoccus, V. uliginosum*, and *V. vitis-idaea*.

* n = 897; 24 logs lacking ground contact at all 7 measuring points are excluded.
